# Combining Urea with Chemical and Biological Amendments Differentially Influences Nitrogen Dynamics in Soil and Wheat Growth

**DOI:** 10.1021/acsomega.4c01451

**Published:** 2024-07-21

**Authors:** Asim Hayat, Ghulam Jilani, Sanaullah Jalil, Tanveer Iqbal, Muhammad Rasheed, Arshad Nawaz Chaudhry, Zeshan Ali, Faisal Zulfiqar, Hayssam M. Ali, Jean Wan Hong Yong

**Affiliations:** † Institute of Soil & Environmental Sciences, PMAS Arid Agriculture University Rawalpindi, Rawalpindi 46000, Pakistan; ‡ LRRI, National Agricultural Research Centre, Islamabad 44000, Pakistan; § Department of Agronomy, College of Agriculture and Biotechnology, Zhejiang University, Hangzhou, Zhejiang 310058, China; ∥ Department of Agronomy, PMAS Arid Agriculture University Rawalpindi, Rawalpindi 46000, Pakistan; ⊥ Ecotoxicology Research Program, Institute of Plant and Environmental Protection, National Agricultural Research Centre, Park Road, P.O. 45500, Islamabad 44000,Pakistan; # Department of Horticultural Sciences, Faculty of Agriculture and Environment, The Islamia University of Bahawalpur, Bahawalpur 63100, Pakistan; ∇ Department of Botany and Microbiology, College of Science, King Saud University, Riyadh 11451, Saudi Arabia; ○ Department of Biosystems and Technology, Swedish University of Agricultural Sciences, Alnarp 23456, Sweden

## Abstract

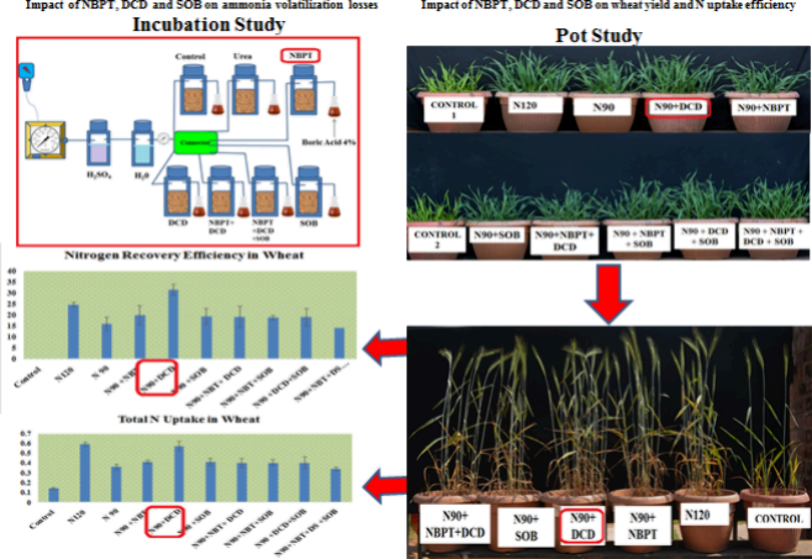

Nitrogen (N) losses from fertilized fields pose a major concern in modern agriculture due to environmental implications. Urease inhibitors, such as *N*-(*n*-butyl) thiophosphoric triamide (NBPT), nitrification inhibitors (NI), like dicyandiamide (DCD), and sulfur-oxidizing bacteria (SOB) could have potential in reducing N losses. For evaluating their effectiveness, investigations were undertaken through incubation and greenhouse experiments by mixing a urea fertilizer with sole NBPT, DCD, and SOB, as well as combined, on ammonia volatilization losses from silt loam soil. An incubation experiment was conducted in 1 L airtight plastic jars with adequate aeration and constant temperature at 25 °C for 10 days. Three replications of each treatment were conducted using a completely randomized designed. The ammonia emission rate gradually increased until the highest (17.21 mg NH_3_ m^–2^ h^–1^) value on the third day with sole urea and some other treatments except NBPT alone, which prolonged the hydrolysis peak until the fifth day with the lowest ammonia emission rate (12.1 mg NH_3_ m^–2^ h^–1^). Although the DCD and SOB treatments reduced ammonia emission, their difference with urea was nonsignificant. Additionally, mixing NBPT with urea exhibited the highest population of nitrifying bacteria in soil, indicating its potential role in promoting the nitrification process. In a greenhouse experiment, 10 treatments, i.e., T_1_ = control, T_2_ = N_120_ (urea fertilizer equivalent to 120 kg N ha^–1^), T_3_ = N_90_ (90 kg N ha^–1^), T_4_ = N_90_ + NBPT, T_5_ = N_90_ + DCD, T_6_ = N_90_ + SOB, T_7_ = N_90_ + NBPT + DCD, T_8_ = N_90_ + NBPT + SOB, T_9_ = N_90_ + DCD + SOB, and T_10_ = N_90_ + NBPT + DCD + SOB, were applied to investigate the wheat yield and N uptake efficiency. The highest N recovery efficiency (31.51%) was recorded in T_5_ where DCD was combined with urea at 90 kg ha^–1^.

## Introduction

Over the past five decades, the global population has experienced an unprecedented increase, nearly doubling its size, leading to a substantial surge in food consumption.^1^ Meeting the projected demands for food security is now of utmost importance, necessitating a significant increase in agricultural production.^2^ However, achieving this heightened productivity has often come at a cost, as it has been heavily reliant on the excessive application of nitrogen (N) fertilizers, leading to imbalanced distribution of applied N in field crops.^3^ Unfortunately, the repercussions of such activities are obvious, with more than half of the applied nitrogen fertilizers in multiple crops leaking to the environment in various forms.^4^ N is a significant plant nutrient that undergoes several transformations in soil, including the generation of gaseous NH_3_ and nitrous oxide (N_2_O).^5^ The loss of nitrogen by emission of gases (N_2_O and NH_3_) from soil reduces the quantity of available nitrogen for crop growth and promotes degradation of the environment via greatly contributing to global warming.^6^

**Figure 1:**
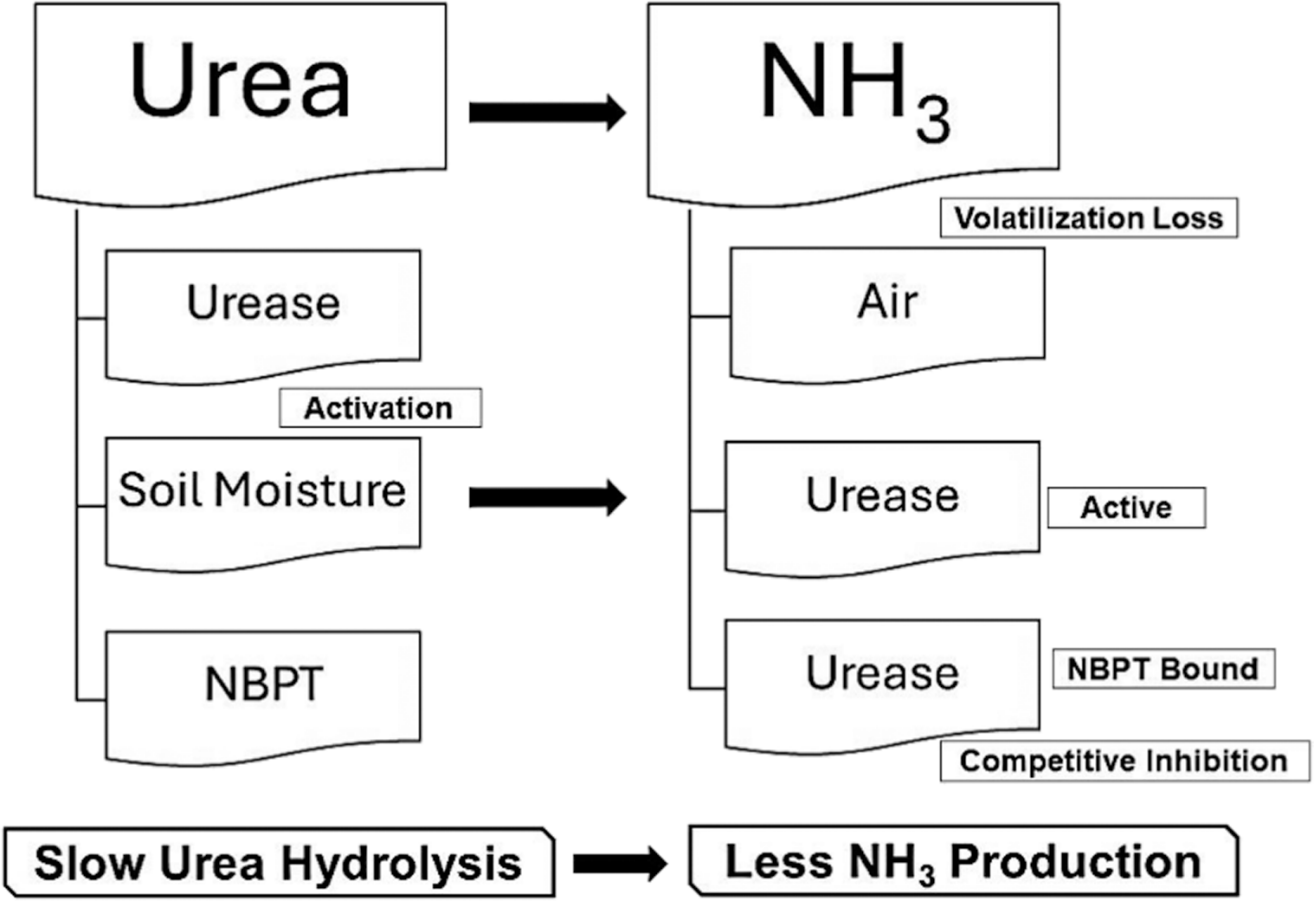
Effect of NBPT on urease activity during urea hydrolysis.

Ammonia volatilization is a critical process that leads to the loss of N from agricultural soils, posing significant challenges to sustainable agriculture and environmental protection.^7^ However, the application of N-based fertilizers, particularly urea, commonly used in modern agriculture, often resulted in substantial ammonia volatilization, leading to reduced nitrogen use efficiency (NUE) and environmental pollution.^8^ Common nitrogenous fertilizers used in agriculture include urea, ammonium nitrate, and ammonium sulfate. These fertilizers considerably affect soil chemical characteristics including, i.e., pH and cation exchange capacity (CEC), consequently influencing NH_3_ volatilization.^9^ The emission of ammonia after nitrogen fertilizer application varies widely depending on soil characteristics such as moisture nitrogen fertilizers, density, and pH, as well as prevailing climatic conditions.^10^ This emission, occurring during and after fertilization, represents a loss of the fertilizer, thereby diminishing its effectiveness and increasing the overall costs of plant production. Typically, the factors influencing ammonia emission are observed to reach their minimum values under conditions of natural pH and low temperatures, while they peak under conditions of high pH and high temperatures. For instance, the emission of NH_3_ per kilogram of applied ammonium nitrate fertilizer converted to nitrogen typically ranges from 16 to 33 g.^11^ Direct application of urea to soil initiates hydrolysis, which occurs through the action of the enzyme urease. This process increases soil pH in the surrounding areas of urea granules, resulting a loss of around 16% of applied N globally through ammonia volatilization (Figure 1).^12^

Under hot and humid climatic conditions, the losses of NH_3_ can reach up to 40% or even higher.^13^ Given the multifaceted challenges posed by ammonia volatilization, there is an urgent need to develop effective mitigation strategies for improving NUE and minimizing its environmental impacts.

In recent years, there has been a growing interest in exploring chemical and biological approaches to mitigate ammonia volatilization from urea in order to promote sustainable and environmentally responsible agricultural practices.^14^ Chemical amendments, such as nitrification inhibitors and urease inhibitors, have shown promise in reducing ammonia volatilization from urea.^14^ Nitrification inhibitors, such as dicyandiamide (DCD) and 3,4-dimethylpyrazole phosphate (DMPP), act by slowing down the conversion of ammonium (NH_4_^+^) to nitrate (NO_3_^–^) through the inhibition of nitrifying bacteria.^15^ Three classes of microorganisms, viz., compound-oxidizing bacteria, ammonia-oxidizing archaea, and ammonia-oxidizing bacteria, are crucial to the nitrification process.^16^ Nitrification inhibitors reduce the availability of NH_4_^+^ for volatilization, effectively retaining N in the soil and increasing its potential for plant uptake.^5^ On the other hand, urease inhibitors, including *N*-(*n*-butyl) thiophosphoric triamide (NBPT), inhibit the activity of the enzyme urease, which is responsible for the hydrolysis of urea into NH_4_^+^ and carbonate ions.^17^ It has been reported that the use of a different inhibitor collectively increased the uptake of N, which in turn increased crop yield as well as NUE with a higher profit rate.^18^ Urease is an enzyme classified under hydrolases that catalyzes the breakdown of urea into ammonia and carbon dioxide. This reaction is significantly accelerated by the enzyme’s presence. Urea hydrolysis occurs in two steps; first, urea is hydrolyzed into ammonium carbonate. In the second step, ammonium carbonate dissociates into ammonium ions and carbon dioxide.^17^
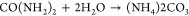
i
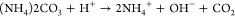
ii

Biological amendments like beneficial soil microorganisms also play a crucial role in N cycling and influence the fate of N compounds in soil.^19^ Certain microbial populations, such as urease-producing and urease-nitrifying bacteria, have the potential to impact ammonia volatilization. Urease-producing bacteria actively participate in the hydrolysis of urea, releasing NH_4_^+^ ions.^20^ However, introduction of microbial strains that competitively utilize urea or modulate urease activity may reduce the rate of NH_4_^+^ release, so effectively mitigating volatilization.^21^ Similarly, nitrifying bacteria are responsible for the conversion of NH_4_^+^ into NO_3_^–^. By introducing microbial strains that compete with nitrifiers for NH_4_^+^ or inhibit their activity, the conversion of NH_4_^+^ to NO_3_^–^ can be slowed down, thereby reducing NO_3_^–^ excess for leaching losses.^22^

Sulfur-oxidizing bacteria (SOB) may also play a crucial role in mitigating ammonia volatilization through a pH reduction in agricultural soils. The SOB possess the unique ability to oxidize sulfur compounds, such as elemental sulfur and sulfides, to sulfate (SO_4_^–2^).^23^ During this oxidation process, SOB release protons, which in turn acidify the surrounding soil environment. This localized acidification increases the retention of NH_4_^+^ in the soil through its conversion to ammonium sulfate (NH_4_)_2_SO_4_, which is less prone to escape as gaseous ammonia.^24^ In addition to soil acidification, the presence of SOB also promotes the formation of stable sulfur–nitrogen bonds, further reducing the potential for ammonia volatilization. These sulfur–nitrogen bonds act as a protective mechanism, preventing the release of ammonia into the atmosphere and preserving nitrogen in a usable form for plants.^25^

In the fertilizer market, innovative inputs such as urease and nitrification inhibitors have gained prominence.^24^ These cutting-edge solutions hold the potential to address critical agricultural challenges by curbing N leaching in the form of nitrate (NO_3_), decreasing NH_3_ emissions, and simultaneously boosting crop yields.^26^ Therefore, the main aim of this research was to quantify the extent of ammonia volatilization losses from soil when applying NBPT and DCD, both with and without the inoculation of SOB to improve the N use efficiency and wheat yield. Additionally, the study sought to explore the correlation between nitrogen losses and the population dynamics of nitrifying bacteria under different amendment conditions.

## 2 Materials and Methods

### 2.1 Incubation Experiments: The Impact of Chemical and Biological Amendments on Ammonia Losses from Soil

#### 2.1.1 Characterization of Soil Samples

An incubation experiment was conducted at the Soil and Environmental Laboratory of Pir Mehr Ali Shah Arid Agriculture University, Rawalpindi, Pakistan. The soil for this study was collected from a 0–20 cm surface layer of a cultivated field, classified as silt loam soil. After collection, the samples were air-dried, ground, and passed through 2 mm sieves and analyzed to determine their chemical and physical properties. Soil pH and EC were measured using a 1:1 (soil:water) ratio with a pH meter (inoLab pH 7110) and an EC meter (inoLab Cond 7110).^27^ Nitrate N and extractable phosphorus contents in the soil were assessed through an ammonium bicarbonate-DTPA extraction method, employing a spectrophotometer (Thermo Fisher Scientific, model no. 51119500). Also, micronutrients (Fe, Mn, Cu, and Zn) and extractable potassium were determined in soil following AB-DTPA extraction using an atomic absorption spectrophotometer (PerkinElmer AAnalyst 800) and a flame photometer (Sherwood, model no. 420), respectively.^28^ Characteristics of soil samples for this study are presented in Table 1.

**Table 1:** Pre-experiment Analysis of Soil for Physicochemical Properties

**variable**	**units**	**mean**	**SD**	**minimum**	**maximum**
pH		8.26	0.03	8.23	8.28
EC	dS m^–1^	0.40	0.01	0.39	0.40
NO_3_	μg g^–1^	0.61	0.09	0.51	0.68
P	μg g^–1^	1.01	0.37	0.71	1.42
K	μg g^–1^	82.00	8.72	76.00	92.00
Cu	μg g^–1^	1.83	0.14	1.67	1.92
Zn	μg g^–1^	2.38	0.18	2.22	2.57
Fe	μg g^–1^	9.95	0.31	9.75	10.31
Mn	μg g^–1^	2.29	0.09	2.21	2.39
sand	%	19.80	0.60	19.20	20.40
silt	%	24.63	0.15	24.50	24.80
clay	%	55.57	0.75	54.80	56.30
textural class	silt clay loam				

#### 2.1.2 Experimental Design and Treatments

**Figure 2:**
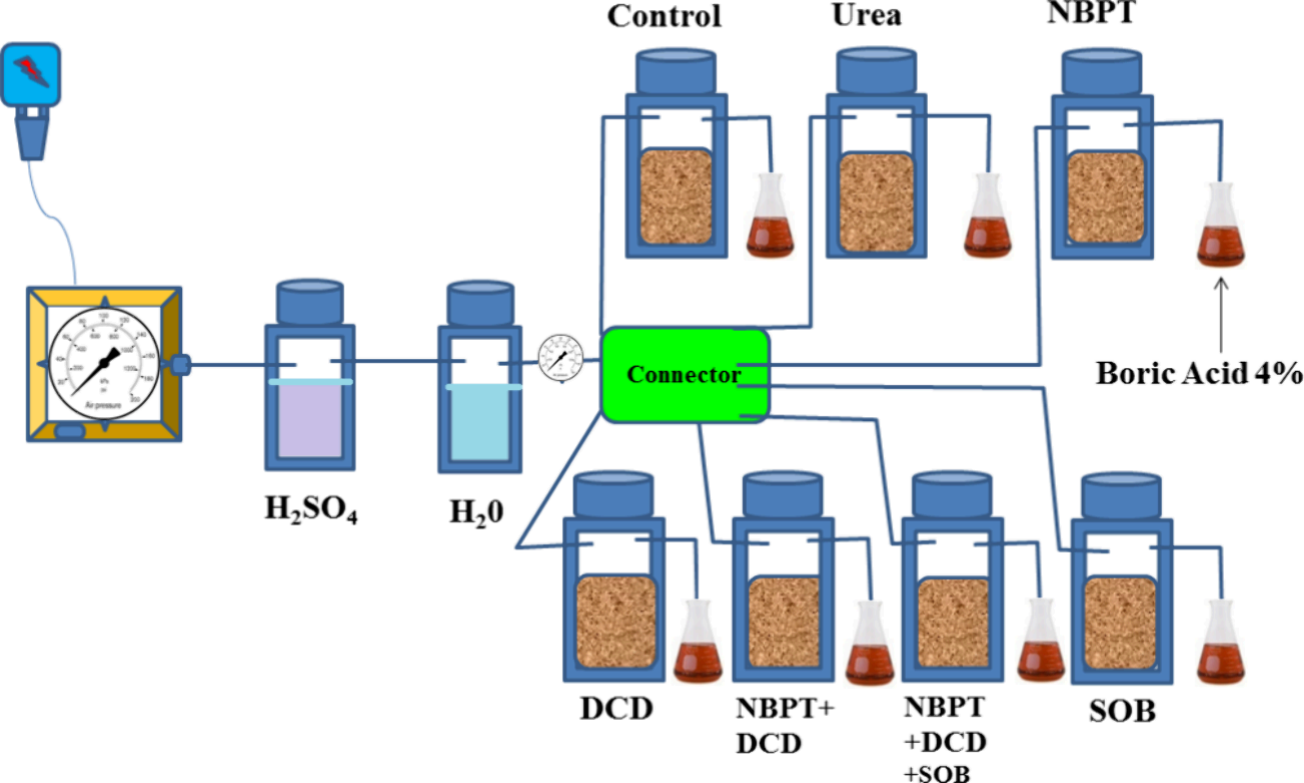
Schematic diagram illustrating the volatilization chamber used for evaluating ammonia emission from soil under controlled conditions.

The experimental setup consisted of plastic jars with a capacity of 1 L and a base area of 20.32 cm^2^, as illustrated in Figure 2. These jars were designed to be airtight from the top, with two small holes (0.5 cm in diameter) located on opposite sides of the walls, and positioned 5 cm below the cap. To facilitate the flow of air into and out of the chambers, plastic pipes of small diameter were connected to these holes. Each plastic jar was filled with 1000 g of finely ground and sieved soil. A constant air flow rate of 1.5 L min^–1^ was maintained throughout the experiment. In order to reactivate the microbial and enzymatic activities, the soil was moistened with water. Air supply to the chambers was provided through an air compressor, maintaining a specific pressure. The air was directed through a solution of 0.1 N H_2_SO_4_ to remove any remaining ammonia followed by passing it through distilled water to preserve soil moisture for microbial activities and prevent rapid desiccation. To prevent mixing between different jars and minimize ammonia losses into the air, all jars were interconnected by using plastic aquarium pipes. Control valves were also installed at the inlet and outlet of each jar to regulate the airflow and maintain appropriate conditions throughout the experiment. The incubation experiment was carried out at room temperature (25 °C). The treatments, following a completely randomized design with three replications, included a control (no amendment), urea at the rate of 60 mg N kg^–1^ of soil (equivalent to 120 kg N ha^–1^), urea at 60 mg N kg^–1^ of soil + NBPT at a concentration of 0.5% w/w N from urea, urea at 60 mg N kg^–1^ of soil + DCD at a concentration of 5% w/w N from urea, urea at 60 mg N kg^–1^ of soil + SOB at 0.1 mL kg^–1^ soil (equivalent to 200 L ha^–1^), urea at 60 mg N kg^–1^ of soil + NBPT + DCD (as in the previous treatments), and urea at 60 mg N kg^–1^ of soil + NBPT + DCD + SOB (as in the previous treatments).

#### 2.1.3 Measurement of Ammonia Volatilization Losses

Ammonia volatilization losses from the soil were quantified using a cylindrical vessel containing 1000 g of soil. The chamber was sealed at the top but had two 0.5 cm-diameter holes on opposite sides, positioned 5 cm below the lid. A constant air flow rate of 1.5 L min^–1^ was maintained using an air compressor, and the pressure was monitored with pressure gauges before the inward flow. The volatilized ammonia was transported through the air and bubbled into a 150 mL conical flask containing a 2% boric acid solution with bromocresol and methyl red indicators. Ammonia was determined by titrating the boric acid solution with 0.1 N H_2_SO_4_.^29^

#### 2.1.4 Determination of the Nitrifier Population

The population of the nitrifying bacteria was measured by the most probable number (MPN) method through serial dilutions and cultivation on specific media. The MPN number of the nitrifier population of the sample was calculated using the MPN table.^30^

### 2.2 Greenhouse Experiments: Response of Chemical and Biological Amendments on Wheat Growth and Nitrogen Dynamics

A pot experiment with three replications was conducted to find the effect of integrated use of inhibitors and SOB on the NUE of wheat crop. The study was executed in the greenhouse of the National Agricultural Research Centre (NARC), Islamabad, Pakistan. The pot dimensions were a surface diameter of 12 in., a bottom diameter of 10 in., and a height of 10 in. A total of 30 pots were utilized, each filled with 10 kg of soil. The soil preparation and characteristics are described in Section 2.1.1. The treatment plan for the pot experiment followed CRD with three replications. Treatments included a control (no N and amendments), N_120_ (equivalent to 120 kg ha^–1^), N_90_ (90 kg ha^–1^ = 75% of the recommended N fertilizer), N_90_ + NBPT (0.5% w/w N), N_90_ + DCD (5% w/w N), N_90_ + SOB (25 L ha^–1^), N_90_ + NBPT + DCD, N_90_ + NBPT + SOB, N_90_ + DCD + SOB, and N_90_ + NBPT + DCD + SOB. The wheat variety chosen for the experiment was Pakistan 2013 (Pedigree; MAX94.27.1.20/3/SOKOLL//ATTILA/3*BCN), developed at the National Agricultural Research Centre (NARC). Five seeds per pot were sown, and the three most healthy seedlings were retained to grow to physiological maturity. During the crop growing season, all standard agronomic practices (weeding, hoeing, thinning, etc.) were followed equally for all treatments. During the crop growth period, the following parameters were recorded.

#### 2.2.1 Chlorophyll Contents

The topmost precleaned expanded flag leaves were selected to record SPAD readings using a calibrated SPAD meter 502. Triplicate readings were recorded from flag leaves of each pot, and average values are reported here.

#### 2.2.2 Phenotypic Traits

The number of tillers and plant height were recorded from each pot, and average values were reported in the study. After harvesting, the wheat plants were tagged, air-dried, and weighed using an analytical balance to obtain a biological yield. The wheat plants from each pot were threshed, and grains were weighed to get grain yield. The crop harvest index was calculated by dividing the economical yield (grain yield) with biological yield (grain yield + straw yield):
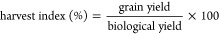
1

#### 2.2.3 Wheat N Uptake

The total N uptake of the wheat plants from experimental pots was calculated using the following equations:
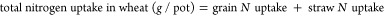
2
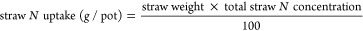
3
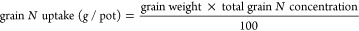
4

#### 2.2.4 Nitrogen Recovery, Agronomic Efficiency, and Partial Factor Productivity

The nitrogen recovery efficiency (NRE) and agronomic efficiency (AE) were calculated through the following formulas:
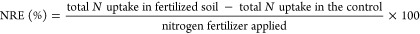
5
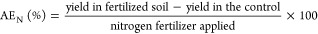
6

The partial factor productivity of applied nitrogen (PFP_N_) was calculated using the following formula:
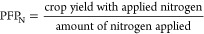
7

#### 2.2.5 Plant Total Nitrogen Content

The total nitrogen content of harvested wheat plant samples (straw and grains) was determined by using the Kjeldahl nitrogen estimation unit (Velp-UDK 149) according to the standard procedure.^31^ The total nitrogen content in the sample was calculated by using the following formula:
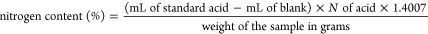
8

### 2.3 Statistical Analysis

Experimental data were analyzed using a completely randomized design through ANOVA, and means were compared by LSD. Statistical analysis was performed by using Statistix 8.1 software at the probability level *p* ≤ 0.05.

## 3 Results

### 3.1 Characteristics of Soil Used for Experiments

The results presented in Table 1 revealed that the soil had a mean composition of 19.8% sand, 24.63% clay, and 55.57% silt, classifying it as a silt loam texture. The mean phosphorus content in the soil was measured at 1.01 μg g^–1^, while the mean nitrogen content was determined to be 0.61 μg g^–1^, indicating deficiencies of these nutrients in the soil. The average value of the electrical conductivity (EC) of the soil samples was 0.40 dS m^–1^, and the mean pH value was found to be 8.26.

### 3.2 Incubation Experiments: The Impact of Chemical and Biological Amendments on Ammonia Losses from Soil

**Figure 3:**
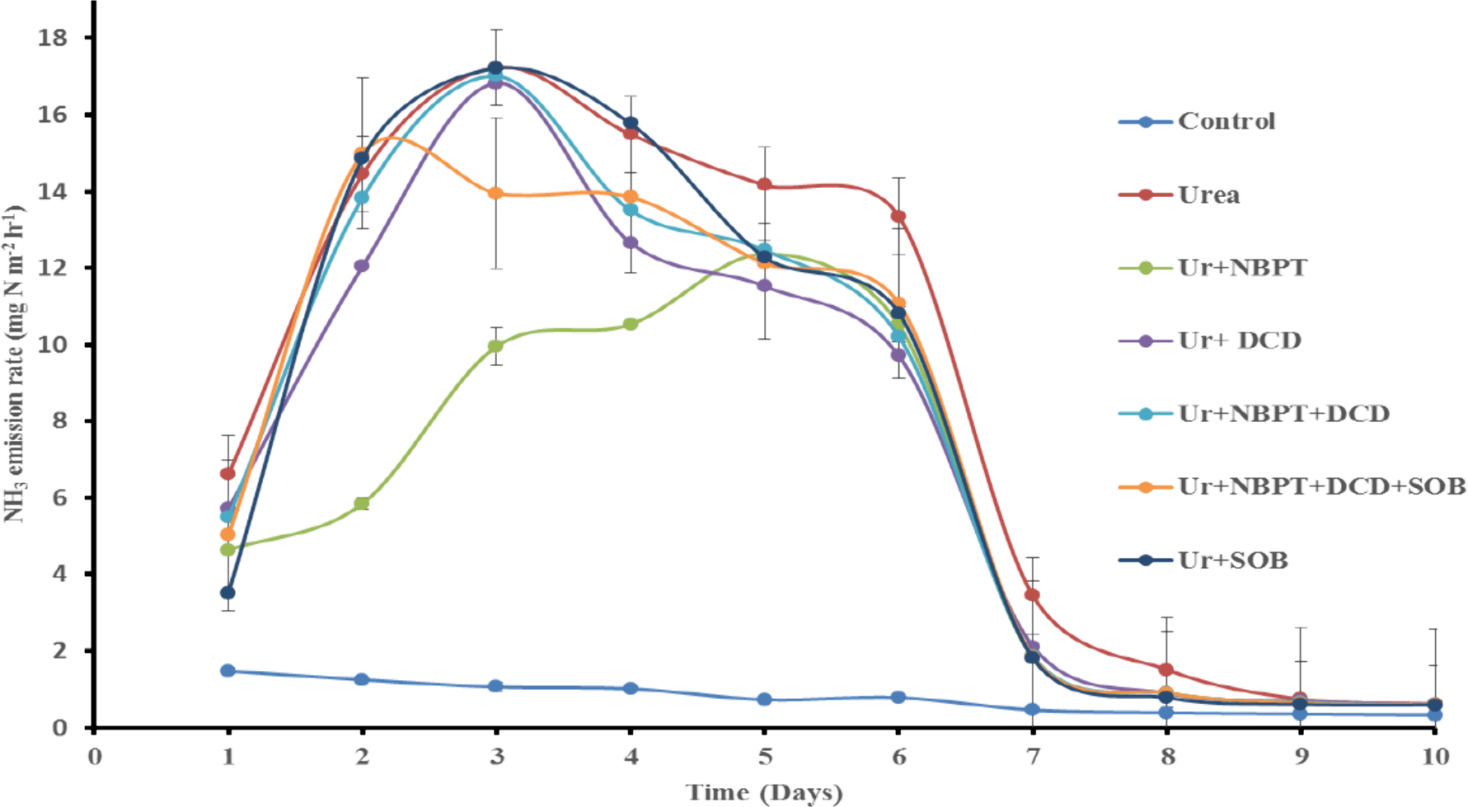
Effect of chemical and biotechnology amendments on the ammonia emission rate.

Figure 3 illustrates the effect of different amendments on the rate of ammonia emission. The lowest ammonia emission was recorded in the control, which did not receive any fertilizer or amendment. Among the treatments that received the N fertilizer, urea + NBPT demonstrated the lowest ammonia release/emission (12.3 mg NH_3_ m^–2^ h^–1^) by delaying the urea hydrolysis process up to the fifth day of incubation compared to other treatments up to the third day. Other treatments released NH_3_ as follows: urea at 120 kg N ha^–1^ > urea + SOB > urea + NBPT + DCD > urea + DCD > urea + NBPT + DCD + SOB, with the maximum values within two to three days of incubation. At the 10th day of incubation, all treatments showed negligible ammonia losses.

**Figure 4:**
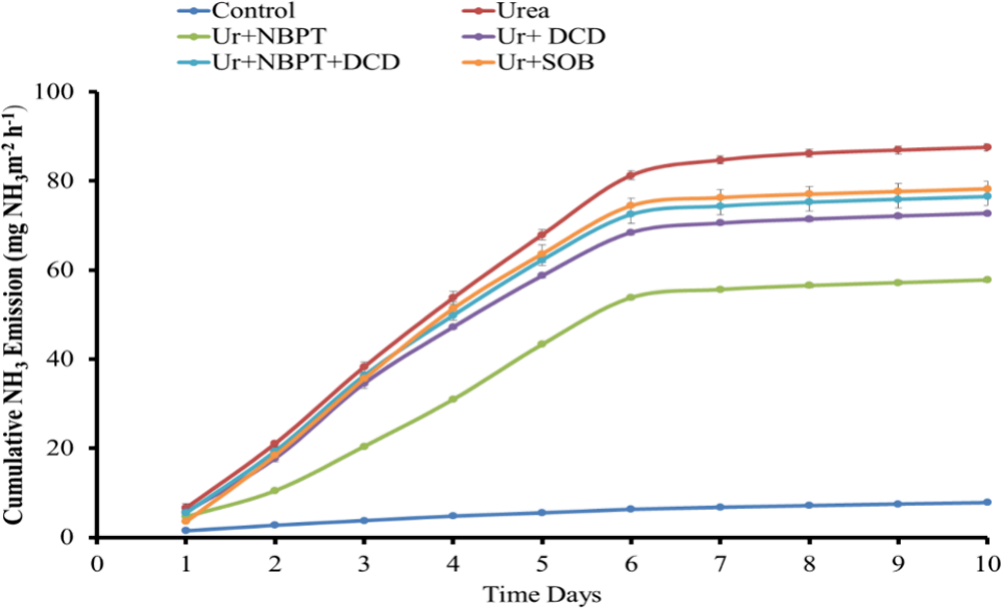
Effect of chemical and biotechnology amendments on cumulative ammonia emission.

Data on cumulative ammonia emission, calculated for different treatments under controlled conditions, are presented in Figure 4. Daily ammonia emission rates were used for the calculations. Results demonstrated that after the control group, the treatment with the lowest cumulative ammonia emission rate (57.8 mg NH_3_ m^–2^ h^–1^) on the 10th day of incubation was the one where urea was applied with a urease inhibitor (urea + NBPT). This was followed by treatment with urea + NBPT + DCD + SOB, which had a cumulative emission rate of 71.2 mg NH_3_ m^–2^ h^–1^. The maximum cumulative ammonia emission rate (87.6 mg NH_3_ m^–2^ h^–1^) was observed in the treatment, where only urea was applied to the soil surface without any amendment. This was followed by treatment with a cumulative emission rate of 78.2 mg NH_3_ m^–2^ h^–1^, where urea was applied with sulfur-oxidizing bacteria. The cumulative ammonia loss at the end of the experiment was higher for treatments without inhibitors compared to urea with the urease inhibitor.

**Figure 5:**
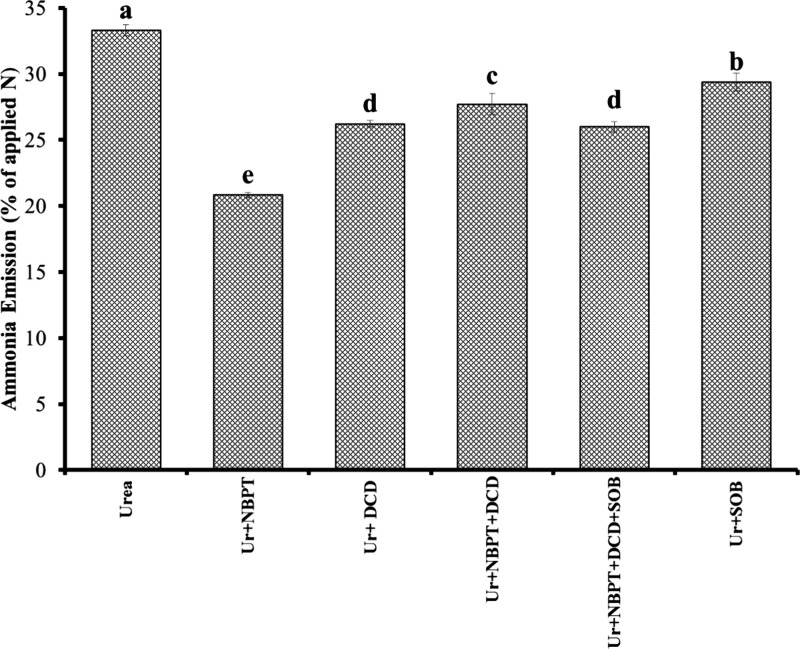
Losses of ammonia from all N sources applied to different treatments. The treatment bars having different letters are significantly different from each other at *p* < 0.05.

Ammonia emission losses with all amendments applied along with urea are illustrated in Figure 5. Urease and nitrification inhibitors contained nitrogen in small quantities, which also contributed during the incubation experiment. These results revealed that NH_3_ losses from urea without inhibitors accounted for 33% of the total applied nitrogen, whereas the treatment with urea + NBPT showed only 21% losses, which were the lowest among all treatments. This was followed by the treatments receiving all amendments as urea + NBPT + DCD + SOB, which showed 26% ammonia losses. The treatment where urea was applied with an inoculum of sulfur-oxidizing bacteria did not show a significant reduction in ammonia emission. Losses were also higher when urea was surface-applied in combination with urease and nitrification inhibitors. The DCD applied alone with urea resulted in higher ammonia emissions compared to NBPT.

**Figure 6:**
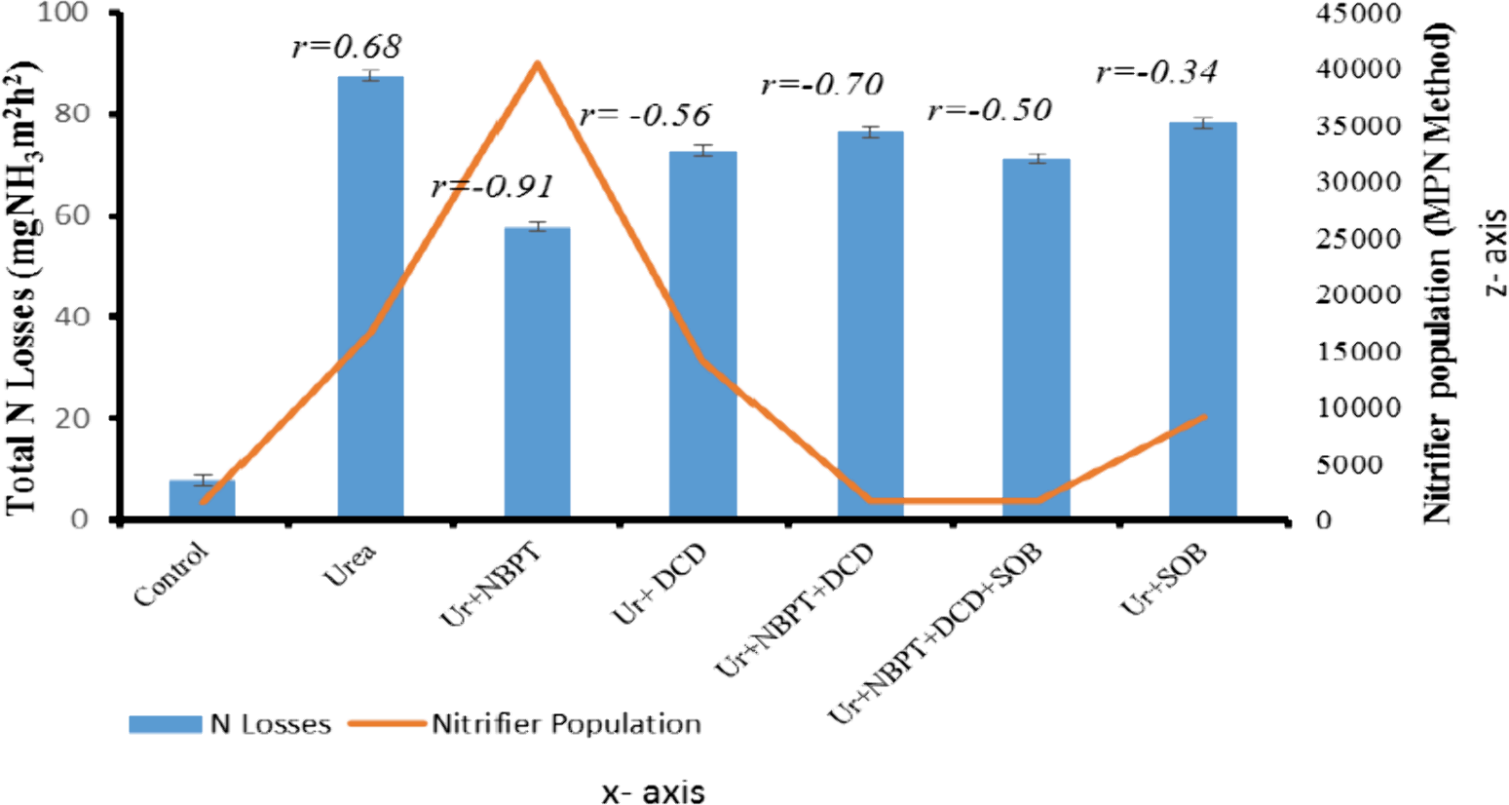
Relationship between the nitrifier population and N losses after incubation.

At the end of incubation experiments, one gram of soil from each experimental jar was subjected to further treatment and incubated for three weeks to establish a correlation between total nitrogen losses and the population dynamics of nitrifying bacteria (*Nitrosomonas* and *Nitrobacter*) using the most probable number (MPN) method. In Figure 6, the red line represents the nitrifier population, while the bar indicates the total nitrogen losses that occurred during the incubation experiment. The graph illustrated a strong negative correlation between nitrogen losses in the treatment, where urea was applied with a urease inhibitor. The highest MPN value (40,500) of nitrifiers was observed in the soil treated with urea + NBPT, while the control group exhibited the lowest nitrifier population (MPN, 1550). Treatments involving the application of urea in combination with urease and nitrification inhibitors, as well as sulfur-oxidizing bacteria, showed lower MPN values compared to other treatments. The treatment where only urea was applied also resulted in a higher MPN value (16,500) followed by the treatment where urea was surface-applied with a nitrification inhibitor (urea + DCD) with an MPN value of 14,050.

### 3.3 Greenhouse Experiments: Response of Chemical and Biological Amendments on Wheat Growth and Nitrogen Dynamics

Results presented in Table 2 indicated that the greatest plant height (30.47 cm) was with treatment where a full recommended dose of nitrogen (120 kg ha^–1^) was applied. It was followed by the plant height (29.68 cm) where 75% of the recommended N (90 kg ha^–1^) was applied with the nitrification inhibitor dicyandiamide (DCD). The smallest plants were seen in the control plot where no nitrogen fertilizer dose was applied. All the amended treatments significantly differed with treatments receiving a full dose of nitrogen and nonsignificantly among each other as far as plant height was concerned.

**Table 2:** Effect of Chemical and Biological Amendments on Growth and Yield Parameters of Wheat^a^

**treatments**	**plant ht. (cm)**	**tillers**	**TBM(g pot**^**–1**^**)**	**grain Y(g pot**^**–1**^**)**	**straw Y(g pot**^**–1**^**)**	**HI %**
control	24.97c	2c	23.8c	10.8d	13d	20.24d
N_120_	30.47a	5a	47.6a	19.53a	33.1a	30.513a
N_90_	27.97ab	4ab	44.47b	14.5c	24.93c	23.343d
N_90_ + NBPT	27.33bc	4.2ab	45.17ab	16.37bc	28.8bc	26.503cd
N_90_ + DCD	29.68ab	4.9a	46.17ab	18.7ab	27.47bc	28.79ab
N_90_ + SOB	29.2ab	3.33bc	43.83b	15.5c	28.33bc	26.117bc
N_90_ + NBPT + DCD	28.43ab	4.33ab	45.47ab	16.53bc	28.93b	26.66bc
N_90_ + NBPT + SOB	29.17ab	4ab	45.33ab	17.1abc	28.23bc	27.357bc
N90 + DCD + SOB	28.37ab	4.33ab	45.13ab	15.57c	29.57ab	25.557 cd
N_90_ + NBPT + DCD + SOB	28.167ab	4.12ab	44.5b	15.03c	29.47ab	25.227 cd

a ht. = height, TBM = total biomass, Y = yield, and HI = harvest index.

A higher number of tillers (5) was noticed in the pots where a full dose of urea was applied, and it was followed by the tillers in treatment where a low dose of N was applied with the DCD inhibitor (4.9). A minimum number of tillers (2) was obtained in the control. There is no significant difference in tillers where a combination of urease and the nitrification inhibitor was applied with 75% of the recommended dose of N. Sulfur-oxidizing bacteria did not show any significant effect.

Results obtained on total plant biomass of wheat as affected by the application of a chemical inhibitor and SOB with treated and untreated urea are presented in Table 2. Results indicated that the maximum total biomass (47.6 g pot^–1^) was with a full dose of urea, but the results of treatment receiving the DCD inhibitor along with urea were also at par. The lowest total biomass was observed in the control (23.8 g pot^–1^) where no N fertilizer was applied. The urease inhibitor NBPT alone and its combination with DCD and SOB did not show significant difference with each other.

The highest grain yield (19.53 g pot^–1^) was obtained in treatment where the recommended dose of N (120 kg ha^–1^) was applied, but a significantly similar yield (18.7 g pot^–1^) was obtained in the treatment receiving a 75% N dose (N_90_) incorporated with DCD (Table 2). The lowest grain yield (10.8 g pot^–1^) was obtained in the control, which is significantly lower than all treatments followed by the treatment receiving only 75% of the recommended dose (N_90_) without any amendment. The treatment where all the amendments were incorporated with 75% of the recommended N showed a significant increase in grain yield compared to sole 75% N treatment. In the case of straw yield, all the treatments except the control and the full dose of recommended N showed a statistically nonsignificant difference with each other.

The effect of the N inhibitor and sulfur-oxidizing bacteria alone or in combination on grain and straw N contents, N uptake, agronomic efficiency, and nitrogen recovery is presented in Table 3. The highest grain and straw nitrogen contents (1.83 and 0.93%) were obtained in treatment where urea was surface-applied in the full recommended dose of nitrogen followed by the greater grain and straw N contents (1.75 and 0.89%) obtained in treatment with 75% of the recommended dose amended with DCD. Treatments where NBPT was applied alone or in combination with DCD gave lower N contents as compared to the treatment receiving DCD as a nitrification inhibitor along with urea. Lower N contents were observed where all amendments were applied in combination compared to DCD and NBPT alone. The lowest grain and straw N contents (0.97 and 0.3%) were obtained in the control.

**Table 3:** Effect of Chemical and Biological Amendments on N Uptake and NRE of Wheat^a^

**treatments**	**grain N con. (%)**	**straw N con. (%)**	**straw N uptake(g pot**^**–1**^**)**	**grain N uptake(g pot**^**–1**^**)**	**total N uptake(g pot**^**–1**^**)**	**agronomic efficiency**	**N recovery efficiency**
control	0.97d	0.3d	0.04d	0.11c	0.14d		
N_120_	1.83a	0.93a	0.23ab	0.36a	0.59a	4.87ab	24.76b
N_90_	1.43c	0.47bcd	0.15c	0.21b	0.36bc	2.73c	15.95cd
N_90_ + NBPT	1.4c	0.65b	0.19abc	0.23b	0.41b	4.13abc	19.93bc
N_90_ + DCD	1.75ab	0.89a	0.24a	0.33a	0.57a	5.87a	31.51a
N_90_ + SOB	1.55bc	0.59bc	0.16c	0.24b	0.41bc	3.5bc	19.34c
N_90_ + NBPT + DCD	1.33c	0.63bc	0.18abc	0.22b	0.4bc	4.27abc	19.05cd
N_90_ + NBPT + SOB	1.45c	0.53bc	0.15c	0.25b	0.4bc	4.67ab	18.84cd
N90 + DCD + SOB	1.44c	0.58bc	0.18bc	0.23b	0.4bc	3.53bc	19.01cd
N_90_ + NBPT + DCD + SOB	1.35c	0.45 cd	0.13c	0.21b	0.34c	3.13bc	14.09d

a N con. = nitrogen concentration.

Statistically higher straw N uptake (0.24 g pot^–1^) was obtained in the treatment where the application rate of nitrogen was 90 kg ha^–1^ along with DCD at the rate of 5% w/w N. It was followed by the treatment with N uptake (0.23 g pot^–1^) receiving the recommended nitrogen dose without incorporation of any amendment. Lower straw nitrogen uptake was recorded in treatments where the N inhibitor and the SOB inoculant were applied together. The lowest straw N uptake was recorded in the control treatment.

In grains, the highest N uptake was recorded (0.36 g pot^–1^) where only urea was applied at the recommended dose of N, being followed by the grain N uptake (0.33 g pot^–1^) in the treatment where DCD was applied along with 75% of the recommended N dose (Table 3). Again, the lowest grain N uptake was observed in the control treatment. On the average, the urease inhibitor alone or mixed with nitrification inhibitor and SOB treatments showed statistically no difference among each other. The total N uptake was higher in treatment where N was applied at the rate of 120 kg ha^–1^ followed by the treatment where DCD was applied alone along with urea at the rate of 90 kg ha^–1^. The lowest total N uptake by wheat plants was recorded in the control where no nitrogen was applied.

The agronomic efficiency (5.87) was greater in the treatment where the nitrification inhibitor DCD was applied along with 75% of the recommended dose of N fertilizer (Table 3). Next, a higher value of agronomic efficiency (4.87) was noticed in the treatment where no N inhibitor was applied, but the recommended N dose was used. The lowest agronomic efficiency (2.73) over the control was observed in treatment where only 75% of the recommended N dose was applied without any amendment.

Table 3 shows that the highest nitrogen recovery efficiency (31.51) over the control was where urea at 90 kg ha^–1^ was applied with DCD at the rate of 5% w/w N fertilizer urea. Treatment with both N inhibitors (DCD and NBPT) and SOB applied with urea at a rate of 75% of the recommended N (90 kg ha^–1^) showed the lowest N recovery efficiency (14.09). It was followed by the next lower N recovery efficiency (15.95) in the treatment where N was applied at 120 kg ha^–1^ without any amendment. The lower nitrogen recovery efficiency in both these treatments reflected the higher amount of nitrogen loss. There was no statistical difference in N recovery efficiency where SOB was applied alone or together with DCD and NBPT mixed in 75% of the recommended N fertilizer urea.

## 4 Discussion

### 4.1 Incubation Experiments: The Impact of Chemical and Biological Amendments on Ammonia Losses from Soil

Various studies conducted across the globe have demonstrated that when urea is applied to the surface of soil, it undergoes significant ammonia losses.^29^ Addition of a urease inhibitor, specifically NBPT, may reduce ammonia losses by delaying the hydrolysis of the urea fertilizer, thereby decreasing ammonia emissions by an average of 60%.^32^ These ammonia emissions are most pronounced in the initial three to seven days following fertilizer application.^33^ Findings of the present study are consistent with earlier published research; studies highlight the beneficial effects of nitrification inhibitors and urease on lowering ammonia volatilization. Results of ref (34) reflected that urea when amended with NBPT can reduce ammonia volatilization losses up to 52%. Addition of the nitrification inhibitor DCD and sulfur-oxidizing bacteria to urea separately has been shown to increase nitrogen losses due to NH_3_ emission.^35^ However, when DCD and NBPT are mixed with urea as a surface application, it reduces NH_3_ volatilization.^36^ There are some claims from different scientists that DCD can influence the efficiency of NBPT, which was not confirmed in this study. The DCD can affect the efficiency of NBPT by inhibiting urea hydrolysis as there is an abrupt increase in soil pH observed in 1 to 7 days following urea application in combination with NBPT + DCD as compared with urea + NBPT alone.^35^ Several other studies^37−39^ supported our results that application of NBPT with urea significantly reduces ammonia volatilization losses as compared to urea alone.

Data of the incubation experiment reflect that NBPT inhibited the urease enzyme and controlled the ammonia emission immediately after urea application and slowly released the ammonia up to the fifth day of incubation. When urea was amended with DCD, SOB, or a combination of DCD + NBPT, it remarkably increased the ammonia emission within the first three days; rather, their effect was seen after the fourth day of incubation. It was also observed that urea when added with the nitrification inhibitor tends to increase the ammonia emission. This occurred because NH_4_^+^ is available for an extended period of time in the soil. This increase in ammonia emission confirmed the early findings that nitrification inhibitors may increase ammonia emission from 3 to 50% depending upon soil and environmental conditions.^40,41^ The NBPT is known as a urease inhibitor and has the ability to block three active sites of the urease enzyme effectively, thus inhibiting the quick process of urea hydrolysis by enhancing the supply of nitrogen.^29^ Peaks of ammonia emission in Figure 3 indicate that more than 50% of ammonia losses occurred within 3 days of incubation from the jar where urea was surface-applied without any amendment.^42^

Urea when added to soil under aerobic conditions is hydrolyzed by the microbial urease enzyme, which generates ammonia that can be converted to nitrite and nitrate when oxidized by microbial nitrification or lost through volatilization. The nitrifier population was further assessed by using the most probable number method through serial dilutions using one gram of soil from each jar after the completion of incubation. The study’s objective was to establish a relationship between nitrogen losses and the population dynamics of nitrifiers. Through the MPN method, it was revealed that the nitrifier population increased in the treatment jar where urea was amended with NBPT. This happened because NBPT delayed the urea hydrolysis up to 7 days, whereas in other treatments, ammonia losses occurred within three days. A correlation between total nitrogen losses and the population dynamics of nitrifying bacteria (*Nitrosomonas* and *Nitrobacter*) was used as a confirmation test to show slow conversion properties of the urease inhibitor, i.e., NBPT, compared with DCD and SOB. At the end of the incubation study, 1 g of soil was taken from each treated jar to perform nitrate and nitrite tests through serial dilutions to find the population of nitrifiers using the most probable number (MPN) method. The correlation was performed at the end of the incubation experiment, which indicated higher population dynamics of *Nitrosomonas* and *Nitrobacter* in the soil where urea was amended with NBPT as compared to the soils treated with DCD and SOB along with urea. It was due to the presence of ammonium in the soil that has been used as a substrate for converting it into nitrite and then nitrate; hence, their population increased in the NBPT treatment at the end of incubation with low N losses.^43^ Moreover our results are in line with the study in ref (44) where due to the inhibitory nature of NBPT, minimum NH_4_^+^ supply was available up to the end of incubation, resulting in little or no effect on the nitrifier population during the whole incubation period.

### 4.2 Greenhouse Experiments: Response of Chemical and Biological Amendments on Wheat Growth and Nitrogen Dynamics

Greenhouse experiments assessed the effects of urease/nitrification inhibitors, viz., NBPT, DCD, and sulfur-oxidizing bacteria, on nitrogen losses, as well as on the growth and yield of wheat. Nitrogen has an important role in vegetative growth of wheat, but it undergoes different losses when applied to the soil. Data obtained from the greenhouse study revealed that a reduced dose of urea (90 kg ha^–1^) amended with DCD significantly improved growth and yield attributes by reducing nitrogen losses. On the other hand, NBPT and SOB alone or in combination did not perform remarkably for improving growth and yield of wheat under silt clay loam soil. The effect of different slow-release fertilizers and DCD with a reduced N rate was studied on wheat under field conditions, and it was revealed that DCD performed better with a 35–37% decrease in N rates.^45^ The DCD was an adequate N control strategy that improved nitrogen efficiency, raised wheat yield, and reduced apparent N losses and ultimately improved economic benefits. Recent research findings concluded that the supply of DCD delayed the conversion of ammonium N to nitrate N and enhanced NH_3_ emission but reduced nitrous oxide emission by 31.4% and significantly increased the yield of maize by 21.3%.^46^

The DCD responded well in the greenhouse by improving the number of productive tillers of wheat, grain yield, and plant biomass when applied with 90 kg N ha^–1^ as compared to treatments receiving a full N dose or a 75% N dose with NBPT, DCD, and SOB. Sulfur-oxidizing bacteria when applied with DCD also showed better results by improving agronomic efficiency and crop yield as compared to NBPT. Higher dosages of nitrogen applied without the use of a nitrification inhibitor result in significant nitrogen losses from nitrous oxide emissions, nitrate leaching, and ammonia volatilization. A decrease in crop yield and nitrogen utilization efficiency follows from these losses. The results of this investigation are in line with those of an earlier study.^47^

The results of several studies have demonstrated that urease and nitrification inhibitors have good impacts on crop nitrogen uptake and nitrogen utilization efficiency in addition to increasing crop yields. The application of a nitrogen fertilizer containing urease and a nitrification inhibitor improves the bioavailability of nitrogen, leading to higher plant biomass, crop production, and nitrogen uptake efficiency, according to the findings of the research conducted in various agricultural systems.^35^ In the present experiment on wheat, application of a nitrification inhibitor, DCD, at a rate of 5% w/w N along with 75% of the recommended dose of urea (90 kg of N ha^–1^) resulted in the highest nitrogen recovery efficiency as compared to the control. Specifically, the nitrogen recovery efficiency was around 60%, which aligns with the experimental findings of another study.^48^ They observed that the application of DCD resulted in improved growth and increased yield for both wheat and maize crops. Notably, in wheat, the nitrogen recovery efficiency improved from 38 to 49%, while in maize, it increased from 27 to 33% when DCD was applied at higher nitrogen levels. Additionally, it was found that DCD was superior to maize in its ability to delay the nitrification process in wheat.

A low dose of urea (90 kg N ha^–1^) combined with DCD recovered maximum nitrogen by reducing N losses and further transformed into grain protein by winter wheat. Different researchers reported that when applied with urea, the urease and nitrification inhibitor improved the efficiency of nitrogen usage by different crops by reducing nitrogen losses.^49^ Compared with the use of different biological and chemical amendments in the greenhouse study, DCD as a nitrification inhibitor applied with urea at the rate of 90 kg N ha^–1^ in wheat significantly improved growth, crop yield, and N recovery efficiency.^50^ Another study reported that nitrification inhibitors (DCD and DMPP) had a significant impact on the soil inorganic nitrogen content.^24^ Specifically, it resulted in a shift in the primary form of soil inorganic nitrogen from nitrate to ammonium. Furthermore, DCD application elevated the concentration of dissolved organic carbon, enhanced above-ground biomass, increased crop yield, and promoted nitrogen uptake by above-ground plants. Our findings are also in line with ref (51) in which authors concluded that application of DCD enhanced growth and yield in both crops. DCD increased the nitrogen use efficiency (NUE) from 38 to 49% in wheat and from 27 to 33% in maize at higher nitrogen levels. Notably, DCD was more effective in slowing the nitrification process in wheat compared to maize.

## Conclusions

The findings of both the incubation and greenhouse experiments disclose the importance of using chemical and biological amendments to optimize the nitrogen use efficiency and mitigate nitrogen losses in soil. In the incubation study, it was evident that the urease inhibitor NBPT effectively controlled ammonia emissions by delaying urea hydrolysis. Among different treatments, urea amended with NBPT showed the lowest ammonia release/emission (12.3 mg NH_3_ m^–2^ h^–1^) at the third day of incubation as compared to urea alone that released the highest ammonia emission (17.2 mg NH_3_ m^–2^ h^–1^) on the same day. Meanwhile, at the 10th day of incubation, the lowest cumulative ammonia emission rate (57.8 mg NH_3_ m^–2^ h^–1^) was observed in the treatment where urea was amended with NBPT. The correlation was drawn between total nitrogen losses and the population dynamics of nitrifying bacteria (*Nitrosomonas* and *Nitrobacter*) through serial dilution using the most probable number (MPN). The highest MPN value (40,500) of nitrifiers was observed in the soil treated with urea + NBPT as compared to those of DCD and SOB amendments. The addition of the nitrification inhibitor DCD and sulfur-oxidizing bacteria led to increased nitrogen losses. However, when DCD and NBPT were combined with urea, it resulted in reduced ammonia volatilization, highlighting the synergistic effect of these amendments on minimizing nitrogen losses. The greenhouse experiment demonstrated that a reduced dose of urea (75% of the recommended dose of N) amended with DCD significantly improved wheat growth and yield attributes by reducing nitrogen losses, thereby showing the highest N recovery efficiency (31.51%) as compared to NBPT and SOB alone or in combination. These results emphasize the importance of choosing the right combination of chemical and biological amendments for optimizing nitrogen use efficiency. Further research and field trials are warranted to validate these findings across different agro-ecosystems and soil types to develop practical recommendations for the sustainable management of nitrogen fertilizers in agriculture.

## Data Availability

All data obtained have been included into the manuscript.

## List of abbreviations

AV: ammonia volatilization
CEC: cation exchange capacity
DCD: dicyandiamide
DMPP: dimethylpyrazole phosphate
N: nitrogen
N_2_O: nitrous oxide
NBPT: *N*-(*n*-butyl) thiophosphoric triamide
NI: nitrification inhibitors
NUE: nitrogen use efficiency
SOB: sulfur-oxidizing bacteria
